# Toward integrated and sustainable prevention against diabetes in rural China: study rationale and protocol of eCROPS

**DOI:** 10.1186/1472-6823-13-28

**Published:** 2013-08-07

**Authors:** Rui Feng, Kaichun Li, Jing Cheng, Shaoyu Xie, Jing Chai, Pingfu Wei, Debin Wang

**Affiliations:** 1 School of Health Services Management, Anhui Medical University, Hefei 230032, China; 2 Lu’an Center for Diseases Prevention and Control, Lu’an City, An’hui province, Lu’an 237000, China

## Abstract

**Background:**

Being an intermediate stage in the development of diabetes, pre-diabetics were estimated as high as 14% to 63% in China and one to three quarters of them will develop into diabetes within 10 years. It is well established that the risk of diabetes progression can be modified substantially and a whole range of proven guidelines, protocols and methodologies are available. Unfortunately, most proven interventions are seldom used in daily practice and this is especially true in resource poor rural China. This project aims at demonstrating that an evolutionary intervention package featuring low cost, integration with routine services, cultural sensitization and self-optimization, is effective and sustainable in preventing diabetes.

**Methods/design:**

This project utilizes a quasi cluster randomized controlled trial and a batched implementation strategy in which villages are recruited in 7 blocks within 7 consecutive years respectively. Block 0 involves 3 villages and provides an opportunity for piloting and refining primitive intervention methodologies and protocols. The following 6 blocks consist of 14 villages each and serve as intervention arm; while all the villages not yet started intervention form the control arm. For each block, measurement happens at baseline and every 12 months (for plasma glucose) or monthly (for body weight and blood pressure) after baseline. These arrangements enable documentation of up to 6 years of consecutive measures and detection of lower incidence of progression into diabetes, improved body max index and blood pressure, and increased service use and involvement in healthy dietary and physical activities among pre-diabetics receiving the experimental intervention compared to themselves at baseline or those in the delayed-intervention control condition.

**Discussion:**

China has a long history of separating disease prevention and treatment systems and there is a clear need to leverages key success factors in a synergetic way toward integrated and sustainable diabetes prevention. This project is owned and managed by local health authorities and utilizes available resources. It introduces a package of long-term incentives, establishes ongoing mechanisms for continuous capacity building and quality improvement, and builds up an operational cycle for catalyzing similar efforts in the local prefecture even throughout rural China.

**Trial registration:**

Current Controlled Trials: ISRCTN66772711.

## Background

Type 2 diabetes mellitus (hereafter referred to as diabetes) has been increasing relentlessly since the second half of the 20th century [1-3]. Over 10% of adults are suffering from the diseases in economically affluent counties, e.g., Saudi Arabia, the USA, Switzerland and Austria. More importantly, the pandemic is increasing and expanding rapidly from affecting primarily the developed nations to afflicting also the developing world. Predicted diabetes will claim up to 366 million people worldwide by 2030 [2]. It was believed that diabetes prevalence is relatively low in China [1]. Yet a recent nationwide investigation revealed that age-standardized prevalence of total diabetes in China is also as alarmingly high as 9.7% with about 3% difference between urban and rural residents (11.4% vs. 8.2%) [4]. And it was predicted that the number of patients with diabetes in China will double by 2030 [2]. Diabetes interacts with other major risk factors (e.g., hypertension, dyslipidemia) and increases the risk of a variety of morbidities (e.g., chronic kidney disease, end-stage renal disease, atherosclerosis, coronary heart disease and cerebral ischemia) leading to tremendous physical, psychological and socioeconomic sufferings and burdens [5].

Pre-diabetes, a lesser degree of hyperglycemia, represents an intermediate stage in the development of diabetes [6,7]. One to three quarters of pre-diabetes develop into diabetes within 10 years of detection [8]. Prevalence of pre-diabetes was estimated at from 10% to 30% worldwide and 14% to 63% in China [4,9,10]. This prevalence rate is closely linked to age, obesity, family diabetes history etc. [4,11-13]. It is well established that the risk of diabetes progression can be modified substantially (by 20 to 60%) regardless of nation and population groups [14-16]. Our systematic literature review identified 26 randomized controlled trials (RCTs) on diabetes prevention in China which also documented over 45% diabetes progression reduction [17-42]. Although the precise mechanism linking pre-diabetes and diabetes is not completely known, commonly recognized factors influencing the progression include age, obesity, physical inactivity, hyperinsulinemia, insulin resistance etc. [8]. So, existing interventions preventing diabetes evolve along three major lines, namely physical activities promotion, dietary modification and glucose-lowering medication. In the 26 RCTs carried out in China, medication was used in 11 and not in 15. Results in reducing progression were no better with medication. And this is consistent with most researches in other countries [14-16]. Screening has always been an integral part of interventions against progression from pre-diabetes to diabetes being used to identify priority groups to leverage lifestyle modification and compliance with interventions [10]. In addition, quite a few articles documented successful application of modern information technologies in diabetes prevention and treatment [43]. Encouraged by high efficacies, tremendous efforts have been invested in this regard and a whole range of IECs, guidelines, protocols, tool kits and best practices are available [44].

Unfortunately, most proven interventions are not being fully used in daily diabetes prevention. This is especially true in resource poor rural China where over 75% of the nation’s vast population lives. None of the above mentioned 26 RCTs was launched in rural China. Published and our own preliminary investigations all suggest that rural village doctors seldom involve in diabetes identification and prevention [45,46]. As a result, 42.34% to 81.82% of rural villagers newly screened with diabetes had never been diagnosed as having the disease before [47]. Knowledge about diabetes among rural villagers, especially the elder villagers, was extremely low [48]. A variety of factors may be attributed to this huge gap between proven technologies and application. Firstly, China rural village doctors lack necessary knowledge and skills [49]. Less than 12% of them had ever received formal training on and knew only 43% of basic knowledge about diabetes prevention [50]. Secondly, China rural communities entail various values, norms and costumes that are counteractive to diabetes prevention, e.g., always provide more food than need for guests, drink the most to show friendship, behave quiet to respect others etc. [51,52]. Thirdly, China health system lacks necessary incentives. For example, in Anhui province, only doctors at township or higher levels are allowed to prescribe oral medication or insulin for the treatment of diabetes while village clinic doctors cannot do so. This removal of responsibility for diabetic care also decreases incentives for the village clinic doctors, as the primary caregivers, to be fully alert to the presence of diabetes among their patients or to intervene persistently in those at high risk.

### Aim/objectives

This project aims at devising and demonstrating that an evolutionary intervention package featuring low cost, integration with routine services, cultural sensitization and self-optimization, is effective in preventing diabetes and pre-diabetics receiving the experimental intervention will, compared to themselves at baseline and pre-diabetics in the delayed-intervention control condition show a lower incidence of progression into diabetes, decreased body max index (BMI) and blood pressure, and increased service use and involvement in healthy dietary and physical activities at follow-up. More specifically, the first objective is to devise, implement and evaluate an intervention to reduce progression from pre-diabetes to diabetes among men and women aged 40 years and over (hereafter referred to as 40+) living in rural Anhui. A secondary objective is to establish a sustainable mechanism, in which participating village doctors maintain continuous momentum integrating diabetes prevention with routine medical service ever since initiation of this project and non-participating village doctors have ready mechanisms to learn and joint the project in the future.

## Methods/design

### Project design

This project utilizes a quasi cluster randomized controlled trial using “administrative” villages (referred to as villages) as study units and a batched implementation strategies in which villages are recruited in 7 blocks within 7 consecutive years respectively (Table 1). Block 0 involves 3 villages and provides an opportunity for piloting and refining primitive intervention methodologies and protocols. The following 6 blocks consist of 14 villages each and serve as intervention arm; while all the villages not yet started intervention form the control arm. For each block, measurement happens at baseline and every 12 months (for plasma glucose) or monthly (for body weight and blood pressure) after baseline. These arrangements enable documentation of up to 6 years of consecutive measures for both intervention and non-intervention villages and thus allow for detection of a variety of short to mid-term intervention effects described later in the project monitoring and evaluation (M&E) section and build a block by block feedback and refinement cycle.

**Table 1:** **Block**-**wise villages in intervention years**

**Blocks**	**Participating villages by implementation years**						
	**Year1**	**Year 2**	**Year 3**	**Year 4**	**Year 5**	**Year 6**	**Year 7**
Block0	3	3	3	3	3	3	3
Block1		14	14	14	14	14	14
Block2			14	14	14	14	14
Block3				14	14	14	14
Block4					14	14	14
Block5						14	14
Block6							14
**Total villages**	**3**	**17**	**31**	**45**	**59**	**73**	**87**

### Selection of participants

This project is supported and implemented in Lu’an, one of the largest prefectures in An’hui province. It has 7 counties consisting of 156 townships and 2081 “administrative” villages. The 3 villages included in block 0 were determined upon convenience considerations; while selection of villages into all the remaining blocks employs cluster randomization. More specifically, for each block, 2 villages from each of the 7 existing counties of Lu’an are randomly selected from the complete name list of non-intervention villages of each of the counties. In order to avoid potential contamination, all non-intervention villages with insufficient distance (less than half day walking) to existing intervention villages are excluded from the name list before any new round randomized selection.

Men and women who are 40 and live in the selected villages for over 6 months per year are all eligible for baseline and biannual follow up screening. Those who meet pre-diabetics but exclusion criteria are treated as priority intervention group. Pre-diabetics are defined as villagers with a fasting glucose 5.6 < 6.9 mmol/l who are not currently being treated with oral hypoglycemic medication or insulin. Those with baseline fasting glucose 6.1-6.9 mmol/l but are confirmed by higher level hospital as diabetic and those with a fasting glucose 5.6-6.9 mmol/L but are on treatment with oral hypoglycemic medication or insulin are excluded from the priority intervention group.

All the accredited village doctors from the clinics of the selected villages are encouraged to participate.

### Intervention elements

**Figure 1:**
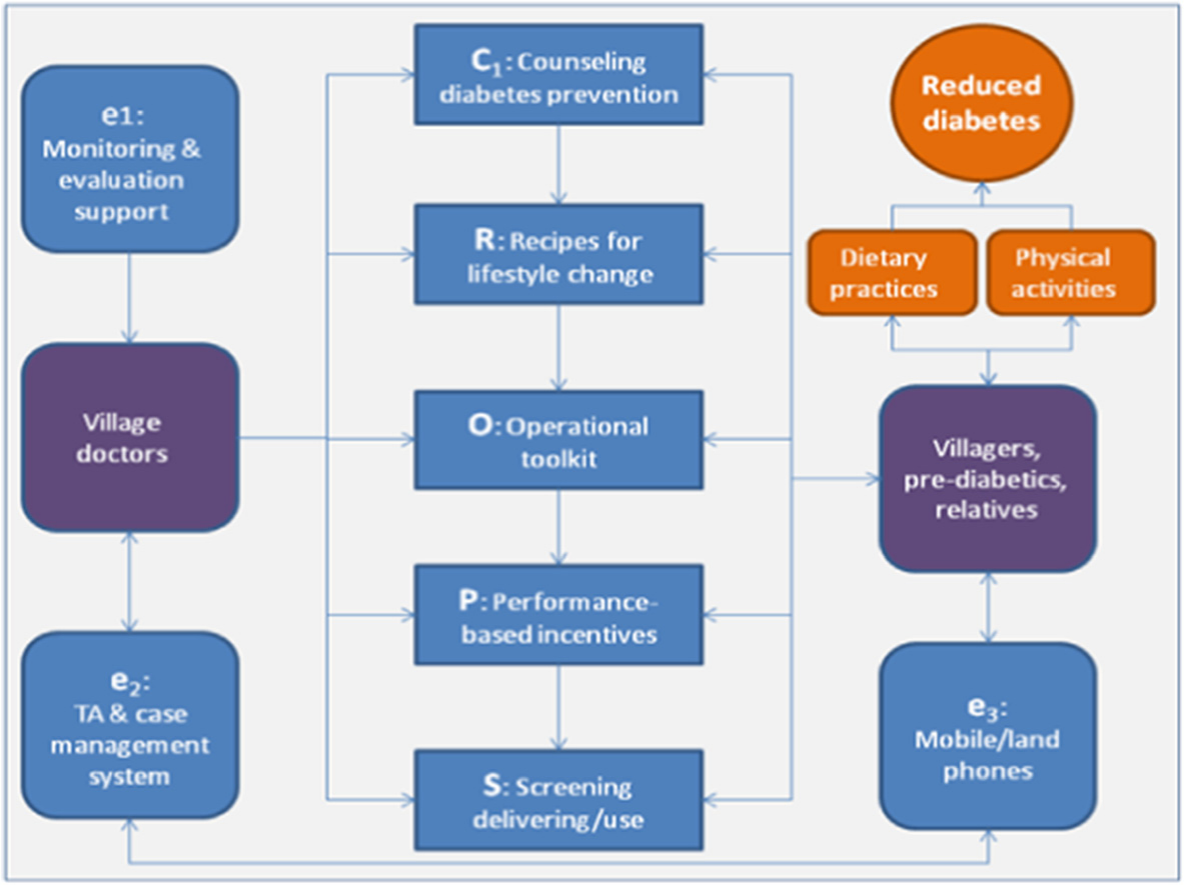
Key components of project intervention (eCROPS).

Given that this project encourages continuous improvement, intervention details may vary from village to village and from time to time. However, key elements of intervention are summarized as eCROPS in which e, C, R, O, P and S stands for educating doctors and electronic supports, counseling diabetes prevention, recipe for lifestyle modification, operational toolkit, performance-based reimbursement for doctors and screening service respectively (see Figure 1).

**Counseling diabetes prevention** (**C**) is delivered by village doctors at village clinics and comprises four kinds of sessions: a) counseling glucose screening applies to 40+ villagers not yet diagnosed with pre-diabetes or diabetes and aims at promoting participation in baseline and biannual follow up glucose screening; b) counseling referrals targets suspected diabetics (fasting glucose >6.1 mmol/l) and urges and helps them to seek higher level diabetes service; c) counseling behavior change planning pertains to newly diagnosed pre-diabetics and uses the newly diagnosed pre-diabetic condition as a powerful trigger to leverage and plan lifestyle changes; d) counseling behavior reinforcement and problem solving starts, for pre-diabetics, one month after the counseling session on behavior change planning at a monthly (in the first year) and bimonthly (in the second year) and quarterly base (in the remaining years) and focuses on reinforcing behavior improvement and solving barriers to behavior changes. All counseling sessions utilizes standard operation procedure (SOP) developed on base of published protocols [53] and tailored via rounds of consensus expert group activities to ensure delivery of minimum key elements, though the counselor village doctors are encouraged to make the best use of their own experiences.

**Recipe for lifestyle changes** (**R**) is a user-friendly “educational wall calendar” for promoting diabetes-related lifestyle modifications. The calendar consists of 12 pages (one for each month). A typical calendar page has 4 pictorial components. On top of the page, is the theme component portraying the key approach and ultimate goal of this project, i.e., “balanced diet and adequate activity improves health and happiness”. The remaining components appear in 3 columns beneath the theme. The left and right column depicts monthly set of tips on how to archive balanced diet and maintain adequate daily physical activity respectively. The middle column divides into 49 cells (7 rows*7columns) each fills with a food picture as background. The top and bottom 8 to 13 of these cells are marked with red “no-parking symbol” on top of food pictures meaning that the foods shown by the background pictures damage health and cause diabetes; while the middle 28 to 31 cells are printed with numbers on top of the colored food pictures indicating, in addition to dates in the corresponding month, that the foods shown in these cells are healthy and help prevent diabetes. Although all the calendar pages share these common components, they differ in color mix, in specific items of recommended and warned foods, and in detailed tips on diet activity changes.

**Operational toolkit** (**O**) gears with a workbook, a manual and a set of cue-cards. The workbook serves as a means for recording minimum information (e.g., date, patient’s name, blood sugar test result, weighing result, summary of counseling, start and end time of the service) about diabetes prevention service delivered by village doctors on encounter by encounter base. The cue-cards are designed to help delivery of key counseling components. In order to facilitate fast selection, the cue-cards are printed in blue, red, yellow and green applying for the four types of counseling mentioned above respectively. Each card enlists critical steps or elements for delivering a specific type of counseling. For example, the yellow card on counseling behavior change planning reminds the following key points: a) trust building; b) implications of pre-diabetic status, its link to diabetes, the seriousness of diabetes; c) the need for immediate lifestyle changes; d) efficacies of lifestyle modifications in preventing progression to diabetes; e) plans to change current dietary and physical habits; f) potential barriers to planned dietary and physical activity changes; g) roles of the pre-diabetic and his or her family members play in implementing the planned changes. The manual is a 3 part user-friendly reference book for village doctors. Part A, elementary protocols, entails step by step SOPs for executing essential prevention tasks, e.g., performing diabetes screening, counseling dietary modification, counseling physical activity planning etc. Part B, common problems and solution tips, gives dos and don’ts to each of the potential problems village doctors often encounter in delivering diabetes interventions, e.g., breaches to planned diet, increasing BMI, failed follow ups, failed attempts refusing over eating at social events etc. Part C, fundamentals of diabetes prevention, provides basic knowledge for effective intervention execution, e.g., pathology of diabetes, counseling principles and techniques, diabetes screening and treatment, socio-cultural aspects of diabetes etc.

**Performance**-**based incentives** (**P**) apply to village doctors and include prevention requirement, financial reimbursement, honor awarding, and membership of Integrated Service Network (ISN). Firstly, the local health authority has issued a policy mandating that all practicing village doctors must deliver a minimum of prevention service so as to qualify future license renewal and participation in diabetes prevention gains required prevention credits. Secondly, diabetes prevention is financially reimbursed upon service volume and quality. Thirdly, each participating village doctor meeting a minimum of diabetes prevention standard will be publicly awarded an honor called Integrative Service Provider (ISP). Fourthly, participation in eCROPS qualifies membership of the ISN that enjoys a) free access to periodical circulation of ISN Newsletter and a web-based forum for sharing cases and experiences maintained by ISN members; b) free enrollment in educational programs offered by ISN that satisfy annual continuing education credits requirement mandated by the local health authorities; c) technical assistance from higher level ISN members including members from township health centers and county, prefecture and province level hospitals; d) dual referral privilege within the ISN. All these incentives are based on performance indicators including mainly plasma glucose, BMI and blood pressure control, and compliance with diet and physical activity plans etc.

**Screening service** (**S**) consists of extended and focused screening and counseling anchored to the screening. Extended screening occurs biannually and covers all villagers aged 40+ but includes only blood sugar testing; while focused screening centers on identified pre-diabetics and includes annual blood sugar and monthly (or bimonthly or quarterly) body weight and blood pressure measurement. These periodical screenings play a key role in leveraging desirable dietary and physical behavior changes for villagers view blood glucose tests, BMI and blood pressure measures as scientific, powerful and convincing. Higher- than-normal results are used to highlight the importance of persistent lifestyle modification. Improving measures are feedback to re-enforce constructive actions already taken by the pre-diabetics. While worsening findings serve as a trigger for looking for and solving potential problems and for promoting more rigorous efforts in dietary and physical activity.

**Electronic supports** (**e**) comprise web-based (built on an ASP.NET plus SQL2008 platform) and user-friendly preventive case management system (PCMS), technical assistance (TA), and monitoring and evaluation support (MES). The PCMS supports village doctors in: a) maintaining a dataset of villagers aged 40+ and participating pre-diabetics; b) scheduling screening and counseling services; c) notifying and reminding (via land or cell-phone) individual villagers about time to attend counseling, weigh-in etc.; d) reviewing performance and intervention history of individual and group pre-diabetics; e) guiding (via built-in standard operating procedures) delivery of major intervention activities e.g., counseling, problem solving etc.; f) entering minimum dataset required by project M&E. The TA for village doctors takes the forms of: a) web-based tutorial on implementing the project prevention in both video and textile formats; b) case studies in which participating village doctors are requested to record and post at least 10% of bottom cases (in terms of compliance with planned lifestyle changes, glucose and BMI control etc.) on the ISN Web-Forum and then experts and other village doctors within the ISN discuss and share experiences coping with similar cases; c) video and pictorial materials about diabetes and its prevention suitable for displaying at village clinics for attending pre-diabetics. The MES is designed for project managers to: a) solicit minimum dataset for project M&E; b) track aggregate and individual deviation from planned objectives and deliverables; c) feedback M&E findings.

### Monitoring and evaluation

The project also emphasizes rigorous yet sustainable M&E closely intertwined with routine prevention services in which data for M&E come mainly from daily service records and findings of M&E are used primarily for improving diabetes prevention.

**Indicators for M**&**E** divide into four categories, namely, socio-demographic characteristics, intervention compliance, equipment measures, and indicator attention score (IAS). Social demographic variables cover age, gender, ethnicity, migration patterns, marital status, education, family income, household membership. Intervention compliance includes: a) the proportion of 40+ year olds who report for screening; b) the proportion of those identified as pre-diabetic who attend at least 10 counseling sessions in the first year of the intervention; and c) the proportion of village clinic doctors who complete the counseling sessions assigned. Equipment measures comprise random and fasting capillary glucose, BMI, systolic blood pressure (SBP) and diastolic blood pressure (DBP). An IAS of a specific village doctor ranges from 0 through to 10 meaning his or her measurement of M&E indicators merits least (IAS=0) to most (IAS=10) attention. It is a composite measure taking into account of: a) last time differences between the “test-retest” findings by the village doctor and a CDC staff separately; b) deviations of last time M&E measures by the village doctor from mean measurements by all village doctors in the same block at the same time point; c) variations in same M&E indicator measured by the same village doctor at the last two time points as compared with that of other village doctors in the same block.

**Time points for data collection** depend on indicator and subjects to be assessed. Random glucose measurement happens at baseline and every two years after intervention initiation in any block for all villagers aged 40+ yet not diagnosed with diabetes or pre-diabetes; while fasting glucose testing occurs at baseline and every year thereafter but covering only participating pre-diabetics or suspected pre-diabetics. SBP/DBP and body weight measurement takes place for all 40+ year participant villagers at baseline and monthly in first year, bimonthly in the second year, and quarterly in the remaining years. Intervention compliance data come from ad hoc prevention service records and data about socio-demographics of pre-diabetes are collected at baseline and renewed annually if necessary. These data all derive from routine prevention service and entered into an online project database by the corresponding village doctors within one week after service delivery. Random “retests” happen at non-fixed-time decided by local CDC staff every year.

**Figure 2:**
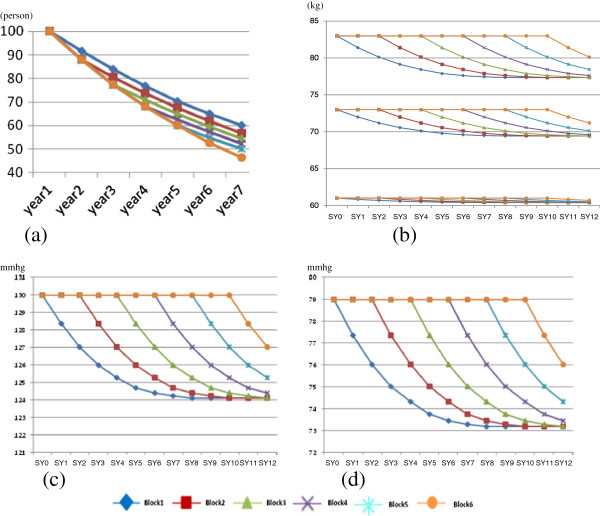
**Expected outcome indicator changes.** Legend: **(a)** expected accumulative survival free of diabetes by implementation blocks and years; **(b)** expected weight changes in different blocks and years; **(c)** expected systolic blood pressure changes by blocks and years; **(d)** expected diastolic blood pressure changes by blocks and years.

**Figure 3:**
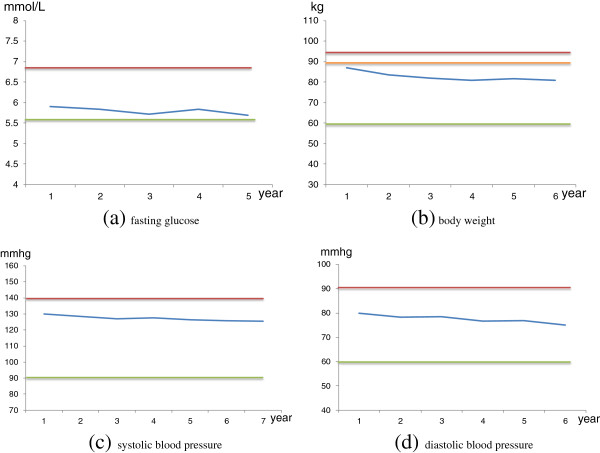
**Example pre**-**diabetics specific outcome plot.** legend: in **(a)**, red line divides pre-diabetes and diabetes, green line divides pre-diabetes and normal and blue line presents individualized fasting glucose; in **(b)**, red line divides obese and overweight, orange line divides overweight and normal, green line divides normal and underweight and blue line presents individualized body weight; in **(c)** and **(d)**, red line divides hypertension and normal blood pressure, green line divides normal blood pressure and hypotension and blue lines presents individualized blood pressure.

**Statistical analysis** centers on two key stakeholders, i.e., managers and researchers, and participating village doctors. Analysis for the former comprises of two steps. Initial analysis consists of descriptive summaries intended to examine major project deliverables and patterns of the various measurements (Figure 2) and check for normality of the continuous variables. And necessary transformations are explored and selected, if necessary, to induce approximate normality. The next step analyses estimates, using two-sided test of the null hypothesis, of the power of differences between the intervention and delayed intervention groups and between different time points in terms of diabetes prevalence rates, accumulated progression rates from pre-diabetics to diabetics, plasma glucose levels, SBP/DBP, BMI, and intervention compliance indicators. For village doctors, analysis produces: a) pre-diabetic specific outcome plots showing the trend in the aforementioned outcome indicators (FPG, BMI, DBP, SBP) compared with that of the normal group (Figure 3); b) village specific performance plots showing the aggregate trend in the outcome and compliance measures of all the participating pre-diabetics within specific villages contrasted with that of all the pre-diabetics in the villages of the same block; c) doctor specific IAS and ranges of top and bottom 25% IASs among all participating village doctors.

**Use of M**&**E findings** serves two closely interrelated purposes, i.e., improving project prevention service and data quality, via a combination of strategies. Firstly, the pre-diabetic specific outcome plots are feedback, on an ad hoc base and via the PCMS, to corresponding village doctors to help them to: a) demonstrate and re-enforce progress made; b) alert lagging behind and leverage more rigorous lifestyle changes; c) identify “difficult” pre-diabetics and set priority intervention targets. Secondly, the village specific performance plots, the IASs and IAS ranges are forwarded twice a year to related village doctors to: a) inform performance achievement compared with peer village doctors so as to generate momentum to maintain or improve performance; b) explore barriers and solutions to higher performance by tracing the lowest performance indicators; c) warn added attention to M&E indicator measurement if the IAS is too high (e.g., greater than that of the top 25 percentile). The project efficacy as well as deliverables, village doctor specific performance indicators and IASs are sent biannually to project management and related CDCs and policy makers to: a) judge overall project progress and insure fulfillment of project milestones and objectives; b) estimate performance-based awards to village doctors; c)arrange additional technical support to poor performer doctors; d) prioritize village doctors (e.g., doctors with bottom 25% IASs) for intensified re-assessment of M&E indicators; e)obtain additional support and adjust the performance-based incentives; f) decide upon project value, continuation, expansion or termination.

## Discussion

Given that China has a long history of separating disease prevention and treatment systems [54], there is a clear need to enhance the health care infrastructure and to teach providers how to help individuals at risk prevent diabetes. The need appears to be most urgent in rural areas, where 75% of the population lives yet lack appropriate resources for curbing the fast growing epidemic. This project strives to tackle a series of barriers to incorporating diabetes intervention, especially behavioral modification with routine medical services. Although with minimum technical support from a research group, this project is owned and managed by local health authorities and utilizes available resources from existing system. This contrasts researcher lead studies that make subtle research (rather than practice) orientation inevitable in almost all aspects and avoids transfer, after project termination, of ownership, capabilities, data etc. More importantly, this project introduces a package of long-term incentives and establishes an ongoing mechanism for continuous capacity building within existing system. These should greatly enhance project sustainability, which is the key to long-term success since diabetes prevention and harm reduction requires lifetime persistence on healthy diet and elevated physical activities. The project also builds up, via the block by block expansion of intervention villages, an operational cycle for catalyzing similar efforts in the local prefecture even throughout rural China.

The second feature of this project worth noting refers to its reliance on village doctors who assume the bulk of project intervention. The large number of village doctors (over one million) in China provides enormous potential for reaching far and wide [55]. As mentioned at the beginning, most village doctors are currently unaware of and certainly not practicing in diabetes prevention [54]. China’s CDC system is staffed with only 190,000 health workers, of whom fewer than 5% belong to the chronic disease prevention taskforce [56]. This project plans to motivate village doctors to work on diabetes prevention for only about 15 minutes per day on average. Even so, if fully implemented in China, this will assume a workload of over 55,000 full-time preventive health workers, over 6 times that of existing taskforce for whole chronic diseases (diabetes is only one among them) prevention in China. There are reasons to believe that village doctors are the best choice for leveraging diabetes prevention for rural villagers. The historical, social and cultural role of village doctors provides necessary professional legitimacy. Chinese people hold strong beliefs that: a) physicians (including village doctors) are respected and trustworthy because they save lives; b) patients should provide their physicians with complete (including confidential) information, for it is in the patient’s own best interest; and c) the human body is complex and patients should take their physicians’ advice as their physicians are educated and experienced in dealing with complex health problems [57]. In addition, village clinics provide an ideal context for diabetes intervention. The physical settings of clinical care are already appropriate for, or can be easily changed to suit, diabetes measurement and counseling. Since most project prevention service can be arranged to coincide routine medical care, the timing of intervention is also important since a patient sees his/her physician only when he/she is ill. This provides a unique teaching moment when health is valued and help from physicians is most needed [58]. Putting together, these can leverage highly effective interventions. Village doctors’ professional knowledge and experience can again greatly facilitate their involvement in diabetes intervention. A minimal amount of training can produce highly effective diabetes interventionists.

Thirdly, this project also relies heavily on electronic supports ranging from telephone messages and educational web pages to computerized case management systems. Although computers and internet are available at almost all village clinics and most village doctors have elementary computer literacy, there are considerable fears about the ability of village doctors and 40+ villagers in rural China to use computerized systems. Therefore, this project emphasizes that each electronic application brings clear added value. For example, the use of web-based training and technical assistance is justified by: a) it saves huge costs compared with traditional training that requires vast village doctors to leave their practices and convey to training facilities from remote rural areas; b) it gives trainees maximum flexibility in choosing training time, pace, format etc.; c) it makes experience sharing an instant access between village doctors working faraway; d) it allows continuous and limitless expansion of trainees; e) through appropriate use of multi-media techniques, e.g., videos and adequate real word piloting, web-based training could be made as effective (if not more effective) as traditional means at a cost much less than that it saves from traditional training.

Fourthly, this project stresses M&E guided continuous improvement. Any project, whatever the design, without adequate M&E is doomed to fail. In this project, M&E informs almost all key decisions ranging from pre-diabetics’ efforts on lifestyle modifications at the bottom to village doctors’ performances in the middle as well as structural changes and project adjustment on the top. The M&E employs the simplest indicators (only six) and measures incurring almost no pain and the least cost for both service providers and receivers. Most importantly, all the indicators are closely linked to the outcomes (health) of the pre-diabetics on one hand and the performance of the village doctors on the other. This clear relationship makes the project M&E most convincing for all stakeholders. The majority of M&E data derive from records of routine prevention by village doctors. This saves measurement costs and enhances seriousness about data recording but also raises concerns about conflict of interests since recording poor cases may result in poorer performance assessment. This conflict is resolved via two mechanisms: a) M&E findings are also used to inform prevention work and accurately recording FPG, BMI etc. helps village doctors in identifying and addressing service weaknesses; b) the minimum random retests and statistical checks will detect data fidelity problems.

Finally, this project leverages key success factors in a synergetic way toward cost- effectiveness and long-term sustainability. For a comprehensive project like this, it is impossible to distinguish the effects of any specific elements except the overall intervention as a whole. Therefore, selecting and combining elements into a working package become vital yet challenging. This project tackles this issue via four major strategies, namely, selection guidance, context tailoring, scrutiny criteria, and soft-systems thinking. In this project, evidences and/or theories guide selection of intervention measures. For example, inclusion of counseling lifestyle changes is based on its efficacy evidences from literature review and design of counseling SOP follows health belief model and motivational interviewing [53,59]. The project targets villagers living in rural China where characterizes strong socio-cultural values and customs and poor resources, capabilities, accessibilities. Tailoring means modifying intervention measures derived from literature and theories according to these local contextual factors. The wall calendar, plots, cue-cards, video format tutorial all result from contextual sensitizations. The project aims at effective, low cost and sustainable interventions and all elements included in the intervention package are subject to rigorous scrutiny against these criteria. The SOPs, for instance, are used on the consideration that they help in ensuring delivery of key contents or steps of prevention service (and hence increase efficacy) and in simplifying service procedures (and hence reduce service delivery and training costs). Soft systems thinking originates from computer system development and views all the elements involved in an interactive and holistic way. Taking the example of random retests for ensuring M&E measurement reliability discussed above, it is added to prevent potential morale risks introduced by the interaction between the performance-based incentives and using routine service records as data source for performance appraisal.

### Human subject protection

This project involves recruitment, intervention and assessment of villagers and village doctors. So it adheres to rigorous human subject protection principles and procedures. The study protocol had been reviewed and approved by the Biomedical Ethics Committee of Anhui Medical University. Participation of villagers and village doctors are 100% voluntary. And written informed consent is sought from all participants.

## Competing interests

The authors declare that they have no competing interests.

## Authors’ contributions

RF and KL contributed equally in conceiving this project, facilitating protocol and SOPs development, and drafting this manuscript. JC (Jing Chai and Jing Cheng) designed the project M&E indicators, measures and use of findings. SX and WF lead the development of performance-based incentives and revised the manuscript critically. DW provided expertise for the overall design of the study, and revised and approved the manuscript.

## Pre-publication history

The pre-publication history for this paper can be accessed here:

http://www.biomedcentral.com/1472-6823/13/28/prepub
